# Longer Nap Duration During Ramadan Observance Positively Impacts 5-m Shuttle Run Test Performance Performed in the Afternoon

**DOI:** 10.3389/fphys.2022.811435

**Published:** 2022-02-09

**Authors:** Omar Boukhris, David W. Hill, Achraf Ammar, Khaled Trabelsi, Hsen Hsouna, Raouf Abdessalem, Nourhen Mezghanni, Nizar Souissi, Nicola Luigi Bragazzi, Karim Chamari, Hamdi Chtourou

**Affiliations:** ^1^Activité Physique, Sport et Santé, UR18JS01, Observatoire National du Sport, Tunis, Tunisia; ^2^Institut Supérieur du Sport et de l’Éducation Physique de Sfax, Université de Sfax, Sfax, Tunisia; ^3^Department of Kinesiology, Health Promotion, and Recreation, University of North Texas, Denton, TX, United States; ^4^Department of Training and Movement Science, Institute of Sport Science, Johannes Gutenberg-University Mainz, Mainz, Germany; ^5^Institute of Sport Science, Otto-von-Guericke University Magdeburg, Magdeburg, Germany; ^6^Interdisciplinary Laboratory in Neurosciences, Physiology and Psychology: Physical Activity, Health and Learning (LINP2), Université Paris Lumières, Paris Nanterre University, Nanterre, France; ^7^Research Laboratory: Education, Motricité, Sport et Santé, EM2S, LR19JS01, Sfax University, Sfax, Tunisia; ^8^Department of Education Collage of Sport Science, Taif University, Taif, Saudi Arabia; ^9^Department of Neuroscience, Rehabilitation, Ophthalmology, Genetics, Maternal and Child Health (DINOGMI), University of Genoa, Genoa, Italy; ^10^Department of Health Sciences (DISSAL), Postgraduate School of Public Health, University of Genoa, Genoa, Italy; ^11^Department of Mathematics and Statistics, Laboratory for Industrial and Applied Mathematics (LIAM), York University, Toronto, ON, Canada; ^12^Aspetar, Orthopaedic and Sports Medicine Hospital, FIFA Medical Centre of Excellence, Doha, Qatar

**Keywords:** nap, Ramadan, sleep, physical performance, perceived exertion

## Abstract

It is well-documented that changes in the rhythm of life during Ramadan affect sleep schedules (i.e., interruption of night sleep patterns) and are likely to have negative effects on physical and cognitive performances. The aim of the present study was to examine the effect of different naps opportunities’ durations during Ramadan on performance of short-duration repetitive maximal exercise and perception of effort. Fifteen physically active men (age: 21 ± 3 years, height: 177 ± 6 cm, body-mass: 73 ± 10 kg) performed a 6 × 30-s shuttle run test after a 25-min nap (N25), a 45-min nap (N45), and in a no-nap condition (NN) during three experimental periods: ∼2 weeks before Ramadan (BR), the last ten days of Ramadan (ER), and ∼3 weeks after Ramadan (AR). During the shuttle run test performed in the late afternoon, the greatest distance (GD), the total distance (TD) and a fatigue index (FI) were assessed. Rating of perceived exertion (RPE) was determined after each 30-s effort. Dietary intake and sleep quality were assessed in each of the three periods. Compared to BR, GD and TD were lower in the ER testing period (*p* = 0.005; *d* = 0.54) but returned to BR levels in the AR period. During ER, carbohydrate intake was lower (*p* = 0.04; *d* = 0.2), and sleep duration and sleep quality were reduced (*d* = 0.27 and 0.54, respectively), although other aspects of dietary intake and sleep pattern were not affected. Compared to NN, GD and TD were higher after N25 (*d* = 0.57 and 0.34, respectively) and N45 (*d* = 0.93 and 0.88 respectively). RPE was lower in N45 (*p* = 0.035, *d* = 0.84). N45 resulted in higher TD (*p* = 0.021, *d* = 0.13) and lower RPE (*p* = 0.004; *d* = 0.57) compared to N25 during ER. Taking a daytime nap benefits subsequent performance in a shuttle run test, whether sleep the previous night was normal (as in BR) or compromised (as in ER). The benefits of napping were greater after a 45-min nap opportunity than after a 25-min nap opportunity.

## Introduction

Healthy Muslims yearly refrain from eating, drinking, smoking, amongst others, from dawn to sunset for 29–30 days during Ramadan observance (Trabelsi et al., 2020a,b). Every day before dawn, Muslim consume a pre-fast meal called “*Sahour or Suhoor*” and then fast until sunset. The breaking fast meal is named “*Iftar*” and eating and drinking are permitted until next dawn. The time lapse between sunset and dawn, and the resulting effects can drastically change according to the location’ latitude and time of the year (Chamari et al., 2019). The obligation to eat only overnight span can lead to several changes in sleep scheduling (Trabelsi et al., 2020a,^2021^) and quality and meal timing and composition (Trabelsi et al., 2020b,^2022^) and previous studies have shown disturbances during the month of Ramadan in the quantity (Leiper et al., 2008; Aziz et al., 2010; Herrera, 2012) and quality (Zerguini et al., 2007; Chamari et al., 2016) of sleep, dietary intake (Trabelsi et al., 2012a,b; Abedelmalek et al., 2015), as well as water consumption (Trabelsi et al., 2012a,b; Aloui et al., 2019).

The importance of sleep for athletic performance is well documented in the literature (Samuels, 2008; Mah et al., 2011; Fullagar et al., 2015; Kölling et al., 2019). Indeed, sleep is a key factor in optimizing training, competitions and recovery (Samuels, 2008; Fullagar et al., 2015; Kölling et al., 2019). Previous studies revealed that napping might reduce fatigue (Brooks and Lack, 2006; Hsouna et al., 2019a) and improve sport (Waterhouse et al., 2007; Blanchfield et al., 2018; Hammouda et al., 2018; Keramidas et al., 2018; O’Donnell et al., 2018; Abdessalem et al., 2019; Boukhris et al., 2019a, 2020a,^2021^; Chtourou et al., 2019a; Daaloul et al., 2019; Hsouna et al., 2019a) and cognitive (Verweij et al., 2016; Daaloul et al., 2019; Hsouna et al., 2019a; Boukhris et al., 2020a) performances. In this context, it has been reported that a nap could reduce fatigue and/or improve vigor, subjective alertness, objective vigilance, and cognitive performance (Brooks and Lack, 2006; Verweij et al., 2016). In addition, Waterhouse et al. (2007) examined the effects of a 30-min post-lunch nap in partially sleep-deprived athletes and showed that 20-m sprint performance was improved with napping. However, Petit et al. (2014) did not find a significant effect of a 20-min nap on peak 5-s power during the Wingate test in athletes after normal sleep or after a simulated jet lag 5-h phase advance sleep. On the other hand, Hammouda et al. (2018) reported that a 20-min or a 90-min nap, taken after partial sleep deprivation, both improved repeated-sprint performance, and that the longer the nap, the greater was the improvement. Similarly, Blanchfield et al. (2018) reported that a short afternoon nap (34 ± 12 min in bed with 20 ± 10 min sleep time) improved endurance performance in runners who had obtained less than 7 h of sleep during the previous night. Moreover, Daaloul et al. (2019) showed that a 30-min nap is an effective strategy to overcome the cognitive and physical deteriorations in performances caused either by sleep loss or by fatigue induced by exhaustive training sessions in the afternoon. A post-lunch nap opportunity has recently been shown as beneficial on physical performance (i.e., jump velocity (O’Donnell et al., 2018), 5-m shuttle run test (Abdessalem et al., 2019; Boukhris et al., 2019a, 2020a,^2021^) and 5 jump test (Hsouna et al., 2019a) and attention (Hsouna et al., 2019a; Boukhris et al., 2020a), with better results observed after a 45-min nap (Boukhris et al., 2019a; Hsouna et al., 2019a) compared to shorter nap durations.

Many studies have examined the impact of the observance of Ramadan on physical performance. Several studies have shown that strength and high-intensity aerobic and anaerobic performances were lower during Ramadan compared to before Ramadan (Chtourou et al., 2011, 2012; Aziz et al., 2012, 2018) and others have reported higher ratings of perceived exertion (RPE) after carrying out a Wingate test or a repeated sprint test (Chtourou et al., 2011, 2012; Hammouda et al., 2012). However, these negative effects are not universal, and other studies have failed to observe substantial performance decrements (Karli et al., 2007; Chaouachi et al., 2008).

As noted above, it has been reported that changes in the rhythm of life during Ramadan mainly affect sleep schedules and dietary regimens (Boukhris et al., 2019b). It is well-documented that interruption of sleep patterns is likely to have negative effects on physical and cognitive performances (Reilly and Waterhouse, 2007; Shephard, 2012) and disturbances of sleep and diet are the main cause of the reported changes in physical performance and behavior during Ramadan (Chamari et al., 2019; Chtourou et al., 2019b). The month of Ramadan is characterized by an increase in daytime sleepiness (Qasrawi et al., 2017). Given that subjective sleepiness can be reduced by taking a nap of 15–45 min during the post-lunch dip (i.e., around 14h00) (Hayashi et al., 1999; Horne and Reyner, 2001), it is not surprising that occurrences of napping increase during the month of Ramadan (Waterhouse et al., 2008). In this context, Roky et al. (2012) indicated that athletes should add some naps during the day during Ramadan to overcome the negative effects of night-time sleep reduction. However, during Ramadan observance, Hsouna et al. (2020) found no significant effects of a 25-min nap on physical performance during the 5-m shuttle run test. The authors potentially attributed this lack of effect could be related to the short nap duration. Certainly, it would behoove physically active Muslims to identify strategies to cope with the interruption of dietary intakes and the disruption of sleep that occur during Ramadan fasting (Chamari et al., 2019). However, to the best of the authors’ knowledge, only one study has examined the effect of a 25-min on physical performance during Ramadan and no previous study has investigated the effect of other daytime nap duration on physical performances during Ramadan.

Therefore, the first purpose of the present study was to investigate the effects of daytime napping on physical performance and effort perception before, during, and after Ramadan. Two durations of nap opportunity were used in the current study. The 25-min duration was used because of the many studies that have reported an improvement in physical performance associated with nap durations of 30 min or less (e.g., Blanchfield et al., 2018; Keramidas et al., 2018; Abdessalem et al., 2019; Daaloul et al., 2019). The 45-min duration was used because of consistent reports of improvements in physical performance when nap durations were higher than 30 min (e.g., Boukhris et al., 2019a; Hsouna et al., 2019a) and because at least one published study has found no positive effect of the shorter nap (Hsouna et al., 2020). We hypothesized that an afternoon nap during Ramadan would have a beneficial effect on short-term maximal performance and on the perception of effort, and that benefits would be greater with a 45-min nap than with a 25-min nap.

In order to explain the increase or decrease of physical performance after napping during Ramadan observance, relationships between RPE and the physical performance during the 5-m shuttle run test should be described. In fact, Boukhris et al. (2019a,2020a) reported that the enhancement in physical performance during the 5-m shuttle run test following daytime nap opportunity was associated with the lower level of RPE recorded during the 5-m shuttle run test. Therefore, the second aim of the present study was to verify if changes in performance were related to RPE before, during, and after Ramadan.

## Materials and Methods

### Participants

The sample size was calculated *a priori* based on procedures suggested by Beck (2013) and using the software G*power (Faul et al., 2007). Values were set at 0.05 for α and 0.95 for power. Based on the studies of Boukhris et al. (2019a,b) and discussions between the authors, effect size was estimated to be 0.65 (medium effect). Required sample size for this study was 10. Fifteen physically active men (age: 21 ± 3 years, height: 177 ± 6 cm, body mass: 73 ± 10 kg) volunteered for this study. The participants were recruited by advertising in classes and posting notices on bulletin boards. After receiving a description of the protocol, potential risks and benefits of the study, participants gave their written consent to participate in this investigation. The criteria for participant inclusion in the present study were as follows: all participants were non-smokers, did not have pathological sleep disorders (i.e., each scored <5 on the Arabic version of the Pittsburgh Sleep Quality Index (PSQI)), and did not consume alcohol. They regularly practiced physical exercise (e.g., walking, jogging) for at least 4 h a week.

The study was conducted according to the Declaration of Helsinki and the protocol was fully approved by the institutional Research Ethics Committee (CPP: 0098/2018) before the commencement of the assessments. The study was carried out in Tunisia in 2016. The month-long observance of Ramadan started on the June 6 and ended on July 5. The time from dawn to sunset, and therefore the length of each day’s fast, was 16h33min. The average temperature and relative humidity during the three periods of data collection of this study were: before Ramadan, 28°C and 50%; during Ramadan, 32°C and 48%; and after Ramadan, 31°C and 47%.

### Experimental Design

**FIGURE 1:**
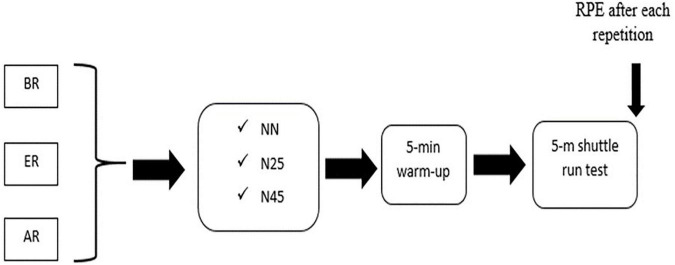
Schematic representation of the experimental protocol. RPE: rating of perceived exertion; BR: ∼2 weeks before Ramadan; ER: last ten days of Ramadan; AR: ∼3 weeks after Ramadan; N25: 25-min nap condition; N45: 45-min nap condition; NN: no nap condition.

Participants performed three 5-m shuttle run tests (Boddington et al., 2001) during each of three testing periods. As shown in Figure 1, the testing periods were scheduled 2 weeks before Ramadan (BR, as baseline session), during the last ten days of Ramadan (ER, to allow sufficient period for the effects of daily sleep disruption to accrue), and 3 weeks after Ramadan (AR, to examine the recovery of performance after Ramadan, as it was previously shown that the effect of Ramadan on sprint performance can persist for at least two weeks AR (Girard and Farooq (2012)). The three shuttle run tests in each testing period were performed under each of three conditions; participants were tested once in a no-nap condition (NN), once after they were provided a 25-min nap opportunity (N25), and once after they were provided a 45-min nap opportunity (N45). Data were analyzed to determine if responses were affected by the observance of Ramadan (effect of testing period), to determine if responses were affected by the opportunity to nap 25 or 45 min (effect of nap condition), and to determine if the effect of napping was influenced by the testing period (nap condition by testing period interaction).

During Ramadan, all participants had not eaten since just before sunrise, in observance of the holy month. During the periods of BR and AR, participants ate a standardized meal before at least 4 h of the test session (Bougard et al., 2009). Furthermore, in the 2 h before the test session, subjects were asked to drink only 500 mL of water to ensure proper hydration (Thorpe and Sunderland, 2012).

Participants completed the Arabic version of the Pittsburgh Sleep Quality Index (PSQI) questionnaire (Suleiman et al., 2010) before, during and after Ramadan. Also, dietary intake was assessed during each period of the study. These data were analyzed to determine if observance of Ramadan affected sleep quality and quantity and/or dietary patterns, which might contribute to any observed decrements in performance during or after Ramadan.

### Testing Procedures

After a familiarization session, participants reported to the laboratory on nine different occasions, separated into three testing periods (BR, ER, AR) with three nap conditions (NN, N25, N45) within each testing period. In each of these nine sessions, participants reported to the testing facility and got into bed at 13h45 in rooms that were favorable to sleep (i.e., dimly lit and quiet with a temperature that typically ranges from 22 to 25°C). After 15 min to become accustomed to the sleep room, participants were asked to either rest quietly for 45 min (i.e., participants were allowed to perform leisure activities such as watching TV, playing video games in a prepared room for these activities) (NN condition), to nap for 25 min (N25), or to nap for 45 min (N45). Daytime napping was realized at 14h00 as this period is taken naturally after lunch, between 13h00 and 16h00, at a time when there is a significant decrease of vigilance and feelings of sleepiness increase strongly (Abdessalem et al., 2019). Abdessalem et al. (2019) compared three nap times (i.e., 13h00, 14h00, and 15h00) and showed that 14h00 and 15h00 were the best nap moments for the 5-m shuttle run test performance improvement. The 14h00 was selected in the present study to allow enough time for participants to avoid sleep inertia. In the current study, the total duration of sleep during napping was not controlled using an objective measurement of the sleep. However, a subjective sleep scale ranging from 0 to 10 was used where 0 indicated “no sleep,” 5 indicated “some sleep with some interruptions,” and 10 indicated “uninterrupted, deep sleep throughout.” After the rest / nap opportunity, participants were asked to move out of the bedroom. They spent the remaining time until 17h00 reading books, watching videos on television, or playing video games in a comfortable armchair. The 5-m shuttle run test (Boddington et al., 2001) was carried out at 17h00 as several studies have shown that Ramadan negatively affects short and long-term athletic performance, especially when the experimental sessions are carried out in the afternoon or evening (Souissi et al., 2007; Chtourou et al., 2012; Hammouda et al., 2012).

During the recovery period after each repetition, the participant provided a RPE and returned to the starting position. The RPE was obtained using an 11-point scale, with scores ranging from 0 (very, very light) to 10 (very, very hard); it has been shown to be a reliable indicator of physical effort, to have sound psychometric properties, and to be strongly correlated with several physiological measures of exertion (Haddad et al., 2013). The average of the six RPE scores was calculated and reported.

Using data from the tests, the following indices were calculated (Boddington et al., 2001; Boukhris et al., 2020b):

1. Greatest distance (GD) (m) = the greatest distance covered in one 30-s shuttle.
2. Total distance (TD) (m) = the sum of distances covered during the six 30-s shuttles.
3. Fatigue index (FI) calculated as follows (Boddington et al., 2001; Boukhris et al., 2020b):

FI(%)=[(shuttle⁢ 1+shuttle⁢ 2)2-(shuttle⁢ 5+shuttle⁢ 6)2]shuttle⁢ 1+shuttle⁢ 22×100

The 5-m shuttle run test was used in the present study as it measures physical performance capacities that are associated with speed and change of direction, and it challenges both the aerobic and anaerobic pathways (Boddington et al., 2001; Boukhris et al., 2019a, 2020b).

In order to assess dietary patterns during each testing period (BR, ER, AR), participants were instructed to accurately record in a diary the estimated quantities of all food and beverages consumed for ten days during each testing period and were interviewed by an experience nutritionist. Dietary records were analyzed using a computerized nutrition system (i.e., Nutrisoft-Bilnut: Food Survey Program version 2.01; France) and the food composition tables of the Tunisian National Institute of Statistics (1978).

The Arabic validated version (Suleiman et al., 2010) of the Pittsburgh Sleep Quality Index (PSQI; Buysse et al., 1989) was used to assess subjective sleep quality over the previous month.

### Statistical Analyses

All statistical tests were processed using STATISTICA Software (Statistica Kernel version10; Stat Software; France). Mean and standard deviation (SD) values were calculated for each variable. The Shapiro-Wilk W-test revealed that values for total energy intake, carbohydrate intake, and total fat intake generated from the information in the diet diaries, and total score generated from responses to the PSQI questionnaire were normally distributed. For these variables, analyses were performed using parametric statistics. Nonparametric tests were used for variables for which the Shapiro-Wilk test was significant and normality could not be established (protein intake from the diet diaries; sleep quality, sleep latency, sleep duration, sleep efficiency, sleep disturbance, and daytime dysfunction from the PSQI; as well as GD, TD, FI, RPE from the shuttle run tests).

To determine the effect of observing Ramadan on dietary intake measures obtained using the diet diaries and on sleep quality measures determined using the PSQI, values from the three testing periods were compared using a one-way repeated-measures analysis of variance (ANOVA) or a Friedman nonparametric ANOVA. When an ANOVA revealed a significant effect, post hoc paired means t-tests were performed to compare pairs of means; when the Friedman nonparametric ANOVA revealed a significant effect, post hoc Wilcoxon tests were performed; in every case the results of paired-means post hoc tests were interpreted using a Bonferroni correction.

To investigate the possible effects of nap opportunity, testing period (effect of observing Ramadan), and the potential interaction between nap opportunity and testing period on data from the shuttle run tests, values from the three nap conditions (NN, N25, and N45) and the three testing periods (BR, ER, and AR) were compared using a two-way ANOVA (or Friedman nonparametric ANOVA) with repeated measures across nap condition and testing period. When the ANOVA results indicated a significant main effect, pairwise post hoc comparisons were performed. When ANOVA results indicated a significant interaction effect, one-way ANOVAs (one across nap conditions and one across testing periods) were performed and, if indicated, these were followed by pairwise comparisons. When the Friedman ANOVA revealed a significant effect, pairwise post hoc comparisons were performed.

To estimate the meaningfulness of significant differences, effect sizes were calculated as partial eta-squared (ηp2) for the normally distributed variables, with values of 0.01, 0.06, and 0.13 representing small, moderate, and large effect sizes, respectively; for variables that were not normally distributed, effect size was estimated by the Kendall’s coefficient of concordance (Field, 2014). To estimate the magnitude of significant differences, percent difference (Δ) scores were calculated as follows:


$$\Delta \left(\%\right)=\left[\frac{\left(\mathrm{Higher}\,\mathrm{value}-\mathrm{Minimum}\,\mathrm{value}\right)}{\mathrm{Higher}\,\mathrm{value}}\right]\times 100$$

To estimate the relationship between performance measures and subjective ratings of the exercise, correlations between the RPE scores and the three performance measures (GD, TD, and FI) were calculated using Spearman’s rank correlation coefficient.

As noted above, significance was accepted for all analyses at the level of *p* < 0.05. Exact *p* values have been given; results given as “0.000” in the statistics output have been reported as “<0.0005”.

## Results

### The Pittsburgh Sleep Quality Index

Results from comparisons of PSQI scores from BR, ER, and AR are presented in Table 1.

**TABLE 1:** Subjective night-time sleep quality recorded before, during and after Ramadan.

	BR	ER	AR	ANOVA
Sleep latency (min)	15 ± 7	17 ± 9	17 ± 6	test = 3.26, *p* = 0.19
Sleep efficiency (%)	96 ± 9	93 ± 8	96 ± 7	test = 5.15, *p* = 0.07
Sleep duration (h)	7.9 ± 1.8*a*	6.7 ± 1.6*b*	7.1 ± 1.6	test = 8.21, *p* = 0.01
Sleep quality	0.9 ± 0.9*a*	2.0 ± 0.8*b*	1.6 ± 0.8*b*	test = 16.33, *p* < 0.0005
Sleep disturbances	0.5 ± 0.5	0.8 ± 0.6	0.7 ± 0.5	test = 6.00, *p* = 0.04
Daytime dysfunction	0.3 ± 0.6*a*	0.9 ± 0.8*b*	0.3 ± 0.5*a*	test = 8.38, *p* = 0.01
Total score of PSQI	3.3 ± 2.5*a*	6.3 ± 2.6*b*	4.8 ± 2.1*ba*	*F* = 22.04, *p* < 0.0005

*a: Significant difference in comparison with ER. b: Significant difference in comparison with BR.*

Compared to BR, during Ramadan (the ER testing period), sleep duration was reduced (effect size = 0.27), daytime dysfunction was increased (effect size = 0.27), and the total score on the PSQI was increased (effect size = 0.61). Three weeks after Ramadan, sleep duration had returned to BR levels; daytime dysfunction had returned to BR levels; and the total score was reduced from during Ramadan but remained elevated above BR levels. Sleep quality was significantly worse during Ramadan (effect size = 0.54) and remained worse through the AR condition.

### Sleep Scale Scores for Naps

All participants were able to fall asleep during each nap condition, before, during and after Ramadan (i.e., a sleep quality score of ∼7 for all conditions; see Table 2).

**TABLE 2:** Sleep scale scores of each nap duration.

	BR		ER		AR	
	N25	N45	N25	N45	N25	N45
Sleep quality scores	7.2 ± 0.9	7.3 ± 0.8	7.3 ± 0.9	7.5 ± 0.8	7.3 ± 0.6	7.3 ± 0.7

### Dietary Intake

Results from comparisons of data from the diet diaries from BR, ER, and AR are presented in Table 3. Total energy intake, total fat intake, and protein intake were constant over the three testing periods. Compared to BR, during Ramadan, carbohydrate was lower during ER (effect size = 0.20).

**TABLE 3:** Estimated daily dietary intake before, during and after Ramadan.

	BR	ER	AR	ANOVA
Total energy intake (kJ/day)	11.15 ± 2	10.50 ± 2	10.25 ± 2	*F* = 2.23, *p* = 0.12
Carbohydrate (g)	339 ± 52	291 ± 70*b*	321 ± 72	*F* = 3.61, *p* = 0.04
Protein intake (g)	84 ± 29	80 ± 20	78 ± 16	test = 0.36, *p* = 0.83
Total fat intake (g)	108 ± 32	114 ± 25	95 ± 26	*F* = 2.97, *p* = 0.06

*b: Significant difference in comparison with BR.*

### 5-m Shuttle Run Test

A Friedman test revealed a significant main effect of experimental conditions on GD (test = 42.54, *p* < 0.0005, Kendall’s W = 0.35), TD (test = 70.14, *p* < 0.0005, Kendall’s W = 0.58) and FI (test = 16.45, *p* = 0.036, Kendall’s W = 0.13).

**FIGURE 2:**
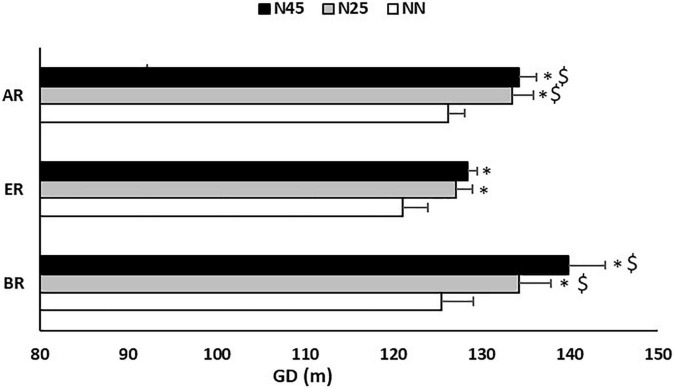
Greatest distance (GD) recorded during the 5-m shuttle run test in the no-nap condition (NN), the 25-min nap condition (N25), and the 45-min nap condition (N45), before (BR), during (ER), and after (AR) Ramadan. *: Significant difference in comparison with NN; $: Significant difference in comparison with ER.

**FIGURE 3:**
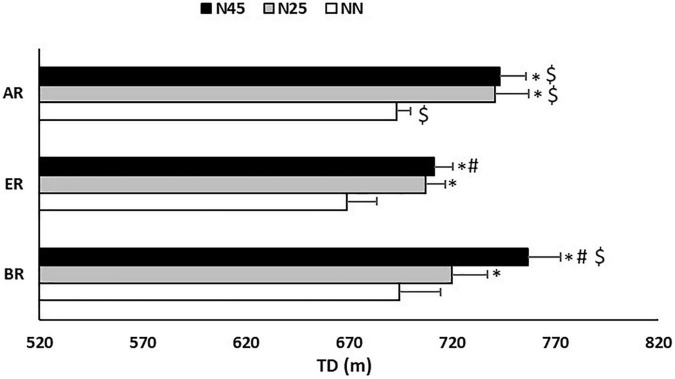
Total distance (TD) recorded during the 5-m shuttle run test in the no-nap condition (NN), the 25-min nap condition (N25), and the 45-min nap condition (N45), before (BR), during (ER), and after (AR) Ramadan. *: Significant difference in comparison with NN; #: Significant difference in comparison with N25; $: Significant difference in comparison with ER.

The pairwise comparisons revealed that the GD (Figure 2) and TD (Figure 3) after NN were 5 and 24 m respectively lower during ER in comparison with AR (GD: Δ = 4.2 ± 4.7%; *p* = 0.005; *d* = 0.54) (TD: Δ = 3.6 ± 4.9%; *p* = 0.005; *d* = 0.54).

Concerning N25, compared to ER, GD was significantly higher 7 m during BR (Δ = 4.8 ± 6.6%; *p* = 0.014; *d* = 0.63). Also, GD and TD were higher by respectively 6 m and 34 m during AR compared to ER (GD: Δ = 4.6 ± 5.7%; *p* = 0.010; *d* = 0.81) (TD: Δ = 4.2 ± 4.7%; *p* = 0.002; *d* = 0.65).

After N45, pairwise comparisons reported that GD was higher by respectively 11 and 6 m during BR (Δ = 7.1 ± 10.9%; *p* = 0.022; *d* = 1.02) and AR (Δ = 4.1 ± 4.8%; *p* = 0.008; *d* = 0.94) than ER, and TD was higher by respectively 45 and 32 m BR (Δ = 5.6 ± 6.6%; *p* = 0.007; *d* = 0.89) and AR (Δ = 4.0 ± 5.5%; *p* = 0.001; *d* = 0.72) than ER.

Regarding the effect of the nap, the statistical analysis showed that during BR, GD and TD were higher by respectively 9 and 25 m in N25 (GD: Δ = 6.3 ± 7.4%; *p* = 0.007; *d* = 0.57) (TD: Δ = 3.6 ± 4.9%; *p* = 0.015; *d* = 0.34) and by respectively 14 and 62 m in N45 (GD: Δ = 9.9 ± 8.6%; *p* = 0.001; *d* = 0.93) (TD: Δ = 8.2 ± 6.4%; *p* = 0.0006; *d* = 0.88) compared to NN. However, compared to N25, N45 resulted in higher TD by 37 m (Δ = 4.9 ± 4.5%; *p* = 0.003; *d* = 0.57).

At ER, GD and TD were higher by respectively 6 and 38 m in N25 (GD: Δ = 4.8 ± 6.0%; *p* = 0.010; *d* = 0.65) (TD: Δ = 5.4 ± 6.2%; *p* = 0.003; *d* = 0.81) and by respectively 7 and 42 m in N45 (GD: Δ = 5.6 ± 8.9%; *p* = 0.014; *d* = 0.85) (TD: Δ = 6.0 ± 5.7%; *p* = 0.001; *d* = 0.93) compared to NN. However, compared to N25, N45 resulted in higher TD by 4 m (Δ = 0.5 ± 7.5%; *p* = 0.021; *d* = 0.13). On the other hands, statistical analysis showed that during ER, FI was lower by 18.3 ± 42.3% in N45 (*p* = 0.030; *d* = 0.84) compared to NN.

**FIGURE 4:**
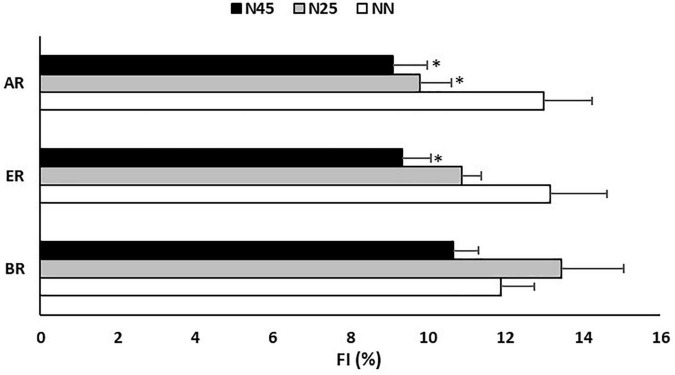
Fatigue index (FI) recorded during the 5-m shuttle run test in the no-nap condition (NN), the 25-min nap condition (N25), and the 45-min nap condition (N45), before (BR), during (ER), and after (AR) Ramadan. *: Significant difference in comparison with NN.

In AR, GD and TD were higher by respectively 7 and 48 m in N25 (GD: Δ = 5.2 ± 6.4%; *p* = 0.012; *d* = 0.92) (TD: Δ = 6.0 ± 6.3%; *p* = 0.003; *d* = 0.98) and by respectively 8 and 50 m in N45 (Δ = 5.8 ± 6.8%; *p* = 0.008; *d* = 1.06) (TD: Δ = 6.5 ± 4.5%; *p* = 0.0006; *d* = 1.25) compared to NN. However, FI was lower by 5.5 ± 82.3% in N25 (*p* = 0.046; *d* = 0.72) and by 12.3 ± 71.1% in N45 (*p* = 0.030; *d* = 0.97) compared to NN (Figure 4).

### Rating of Perceived Exertion During the 5-m Shuttle Run Test

**FIGURE 5:**
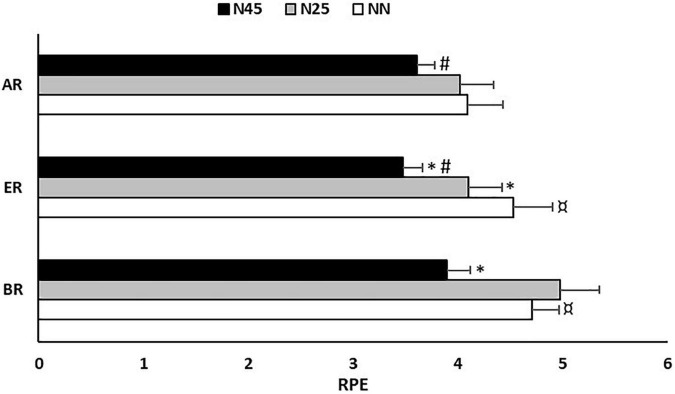
Rating of perceived exertion (RPE) scores recorded during the 5-m shuttle run test in the no-nap condition (NN), the 25-min nap condition (N25), and the 45-min nap condition (N45), before (BR), during (ER), and after (AR) Ramadan. *: Significant difference in comparison with NN; #: Significant difference in comparison with N25; $: Significant difference in comparison with AR.

Results of the Friedman test conducted on RPE mean scores during the 5-m shuttle run test revealed a significant effect of experimental condition (test = 43.08, *p* < 0.0005, Kendall’s W = 0.35). Regarding the effect of the period, the RPE mean scores during the 5-m shuttle run test recorded after NN were higher at BR (Δ = 14.1 ± 12.0%; *p* = 0.004; *d* = 0.51) and ER (Δ = 8.8 ± 11.9%; *p* = 0.005; *d* = 0.28) than AR (Figure 5).

Regarding the effect of the nap, the statistical analysis showed that during the period of BR, RPE mean scores during the 5-m shuttle run test were lower in N45 compared to NN (Δ = –26.4 ± 40.8%; *p* = 0.035; *d* = 0.84). During ER, RPE mean scores during the 5-m shuttle run test were lower by 12 ± 21% in N25 (*p* = 0.028; *d* = 0.28) and by 31.7 ± 35.2% in N45 (*p* = 0.002; *d* = 0.85) compared to NN. Also, RPE mean scores during the 5-m shuttle run test were higher by 17.6 ± 23.7% in N25 (*p* = 0.004; *d* = 0.57) compared to N45. AR, RPE mean scores during the 5-m shuttle run test were lower by 10.1 ± 19.5% in N45 (*p* = 0.029; *d* = 0.39) compared to N25 (Figure 5).

### Correlations Among Performance and Perceptual Values

For N25, there were significant correlations between GD and RPE scores at ER (*r* = –0.62, *p* = 0.012). For N45, there were significant correlations between GD and RPE scores at ER (*r* = –0.55, *p* = 0.032) and AR (*r* = −0.51, *p* = 0.047). In addition to that, for N45, there were significant correlations between TD and RPE scores (*r* = –0.54, *p* = 0.034).

## Discussion

The present study demonstrated that napping improves performance, Ramadan worsens performance, and a nap during Ramadan can abolish the negative effects of Ramadan.

The important finding in the present study is that napping has a positive effect on physical performance (GD and TD) and perceived exertion in a high intensity shuttle run test. Second, the results demonstrate that TD, arguably the most important and robust performance measure, is reduced during Ramadan but returns to BR levels within 21 days of the conclusion of Ramadan. Third, specific to the effects of napping as a counter-measure to the effects of sleep disruption during Ramadan, the results demonstrate that a 45-min nap and, to a lesser degree, a 25-min nap improves the performance measures of GD and TD, and minimizes had a positive effect on the fatigue index (FI), and only during tests carried out during Ramadan.

Sleep patterns have been shown to influence athletic performance, and sleep disruption can negatively affect mood and mental performance (Chtourou et al., 2011; Romdhani et al., 2019). During Ramadan, modifications of training, coupled with the scheduling of meals only during the night, can lead to 1–2 hours of sleep lost per day (Herrera, 2012) and reductions in sleep quality (Aziz et al., 2010; Chamari et al., 2016; Boukhris et al., 2019b). This chronic deprivation of sleep and disruption of sleep-wake cycles induces an excessive daytime sleepiness, fatigue, and lethargy and has a negative impact on mood (BaHammam, 2003; Aziz et al., 2010, 2012), including an increase in total fatigue scores during Ramadan in comparison with the control period (Chaouachi et al., 2009). In addition to the effects on sleep, many studies have also demonstrated reduced performance during Ramadan compared to before Ramadan (Brisswalter et al., 2011; Chtourou et al., 2011, 2012; Hammouda et al., 2012, 2013, 2014; Aloui et al., 2013; Aziz et al., 2018). Despite the challenges of restricting eating and drinking to the hours between sunset and sunrise, the literature reveals that Ramadan fasting *per se* has no adverse effect on dietary intake (Boukhris et al., 2018; Trabelsi et al., 2020a). Thus, impaired physical performance cannot be attributed to the changes in dietary patterns and must be a consequence of the disruption of sleep. Consistent with this hypothesis, several studies have suggested that if sleep characteristics during Ramadan are not different from before Ramadan, the observance of Ramadan will not have adverse effects on short-term high intensity exercise performance (Karli et al., 2007; Chaouachi et al., 2009; Hsouna et al., 2019b).

In the present study, consistent with the results of previous studies (Aziz et al., 2010; Boukhris et al., 2019b), the total score of PSQI, the sleep duration (i.e., 6.7 ± 1.6 h *vs.* 7.9 ± 1.9 h in BR), the sleep quality and the daytime dysfunction were negatively affected during Ramadan in comparison with control periods. With the disruption of sleep came reductions in physical performance. However, although performance in the 5-m shuttle run tests clearly appeared to be negatively impacted across the three nap conditions, the effects of observance of Ramadan in and of itself (i.e., based on responses in the NN condition) were not statistically significant (GD: –4.0 m, –3%, n.s versus BR; TD: –24 m, –3.6%, n.s. versus BR). Despite that lack of statistical significance for the pairwise comparisons, the effect sizes were small (0.39 and 0.38, respectively). This decrease in physical performance is likely due to changes in sleep patterns. It is acknowledged that total caloric intake appeared to be reduced 9% (n.s.) and carbohydrate intake was reduced 14% during Ramadan compared to BR, but it is unlikely that these dietary changes would compromise performance in a 6 × 30 s test.

There are several reports of the benefits of a daytime nap on human performance following partial sleep deprivation (Brooks and Lack, 2006; Waterhouse et al., 2007; Hammouda et al., 2018; Daaloul et al., 2019; Romdhani et al., 2020; Souabni et al., 2021) and following one night of sleep deprivation (O’Connor et al., 2004). These studies reported that naps improve cognitive (Brooks and Lack, 2006), and physical performance (Waterhouse et al., 2007; Hammouda et al., 2018; Daaloul et al., 2019; Romdhani et al., 2020; Souabni et al., 2021), and that they enhance short-term memory and mood (Brooks and Lack, 2006) and reduce subjective sleepiness (Brooks and Lack, 2006; Waterhouse et al., 2007) and fatigue (Brooks and Lack, 2006). These improvements were observed after (i) 20 min nap on repeated sprint performance (Hammouda et al., 2018; Romdhani et al., 2020), (ii) 30 min nap on 2- and 20-m sprints performance (Waterhouse et al., 2007) and endurance performance (Keramidas et al., 2018), and (iii) 90 min nap on repeated sprint performance (Hammouda et al., 2018; Romdhani et al., 2020). Thus, it can be deduced that the naps are a powerful method to improve physical performance when there is a sleep loss. The improvement of performance during the 5-m shuttle run test after an afternoon short-nap during each period could be explained by an improvement in alertness (Brooks and Lack, 2006; Hsouna et al., 2019a) and a reduction in sleepiness (Waterhouse et al., 2007) and subjective fatigue (Brooks and Lack, 2006; Hsouna et al., 2019a). Roky et al. (2004) has contended that the negative effects of observing Ramadan on performance are mediated through sleep disruption, and this may explain why athletes spontaneously nap more during Ramadan compared to BR [i.e., 100 ± 120 *vs.* 10 ± 14 min; (Aziz et al., 2018)]. Logically, napping is a strategy for athletes during Ramadan to overcome the effect of sleep loss and daytime sleepiness.

In the present study, sleep was disrupted during Ramadan (PSQI score increased from 3.3 ± 2.5 to 6.3 ± 2.6) and napping had beneficial effects on all measures of performance in the shuttle run tests. TD, arguably the most important and robust performance measure, reflects contributions of speed, agility, and anaerobic capacity; TD after a 25-min nap (N25 condition) was greater than in the NN condition and TD after a 45-min nap (N45 condition) was greater than in the N25 condition. GD, which reflects contributions of speed, agility, and anaerobic power, was equally improved after the 45-min and 25-min naps compared to the NN value. In contrast with the present results, recently, Hsouna et al. (2020) have failed to observe an improvement in GD and TD after 25-min nap during Ramadan observance. The authors of this study suggested that the lack of significant improvement after a 25-min nap could be related to its short duration. The fatigue index, which is largely based on the values of GD and TD and identifies the ability to maintain high levels of energy production, was improved after a 45-min nap. Values for RPE were progressively smaller from the NN to N25 to N45 conditions. Clearly, napping improved performance. Although it may not be statistically supportable to compare the values, it appeared that napping in Ramadan permitted a TD that was greater than, and certainly no less than, what was accomplished under “normal” or “control” conditions (no-nap, before Ramadan). These results support the contention of Roky et al. (2004) that performance decrements in Ramadan are a consequence of sleep disruption, they are in agreement with studies that have found beneficial effects of napping on performance when there is a sleep loss situation, and they demonstrate that napping is a viable counter measure.

The benefits of napping on human performance following normal sleep have been previously confirmed (Hayashi and Hori, 1998; Abdessalem et al., 2019; Boukhris et al., 2019a, 2020a,^2021^; Hsouna et al., 2019a; Romdhani et al., 2021). These studies reported that naps improve cognitive (Hsouna et al., 2019a; Boukhris et al., 2020a; Romdhani et al., 2021), psychomotor performance (Verweij et al., 2016) and physical performance (Abdessalem et al., 2019; Boukhris et al., 2019a, 2020a,^2021^; Hsouna et al., 2019a; Romdhani et al., 2021), and that they reduce subjective sleepiness (Hayashi and Hori, 1998; Boukhris et al., 2020a,^2021^; Romdhani et al., 2021) and fatigue (Boukhris et al., 2019a, 2020a,^2021^). We note that there have been studies that did not report a benefit to napping in the absence of prior sleep disruption; for example, Petit et al. (2014) reported no improvement in performance with a 20-min nap after a normal night’s sleep. Suppiah et al. (2019) actually reported a significant negative effect of nap (i.e., maximum duration 30 min) after a normal night’s sleep (i.e., 7 h 45 min) on shooting performance, autonomic function (i.e., heart rate variability during simulated 20-min shooting competition), reaction time, and 2-, 10- and 20-m sprint performance after normal sleep. The authors of these two studies suggested that the lack of changes may be attributed to the relatively short nap duration employed in their investigation. Another factor that might influence the effectiveness of napping on performance outcomes is the time elapsed between the end of napping and the subsequent testing (Botonis et al., 2021). Therefore, enough time should be allowed for participants between the end of napping and the beginning of testing to avert the effects of sleep inertia that might have occurred after napping (Botonis et al., 2021). In the present study, more than 2 h was allowed for participants between the nap and the testing, which was enough to overcome any sleep inertia that might have occurred. The difference in napping habituation could be also another factor that affects the results. In this context, it has been reported that non-habitual nappers fall asleep faster and tend to have greater sleep efficiency in comparison with usual nappers (Milner et al., 2006; Petit et al., 2014). In the present study, all the participants were non-habitual nappers.

In the present study, in the BR or “control” testing period, napping had beneficial effects on all measures of performance in the shuttle run tests. TD after a 25-min nap (N25 condition) was greater than in the NN condition and TD after a 45-min nap (N45 condition) was greater than in the N25 condition. GD was equally improved after the 45-min and 25-min naps compared to the NN value. In agreement, Hsouna et al. (2020) showed an increase of GD after a 25-min nap before Ramadan. Values for RPE were smaller in N45 versus NN. Clearly, napping improved performance.

It is a challenge to interpret the results of testing during the AR testing period. Since tests were performed after over 2 weeks of “normal” sleep, diet, and exercise routines AR results arguably could be considered as a second “control” period. Conversely, if the effects of jet lag following one flight across ten time zones has repercussions that persist for 2 weeks, there is every reason to believe that a 30-d change in sleep, diet, and exercise habits may well be expected to have some lingering effects. While some aspects of subjective sleep quality had been restored to normal by the time of the AR testing, the sleep quality score and the total score for PSQI remained elevated above baseline (BR) levels. Sleep duration in AR (7.1 ± 1.6 h) was not different from either the BR or ER values, but mathematically was closer to the ER vale than the BR (Table 1). Coupled with the PSQI data, his suggested a lingering effect of, or a delayed recovery from, observing Ramadan. Yet, all performance measures had returned to baseline levels. Nevertheless, the effects of napping on all four performance measures were obvious in AR, just as in the other conditions. In accordance with the present findings, it has been reported that 25-min nap improved physical performance after Ramadan (Hsouna et al., 2020). Whether the AR testing period reflected delayed effects of Ramadan or re-establishment of baseline conditions, our results clearly demonstrate the effective of an afternoon nap to improve physical performance.

Concerning nap duration, the 45-min nap was superior to the 25-min nap to improve GD and TD and to reduce FI and the RPE scores during the 5-m shuttle run test. The present results are in agreement with previous studies, not carried out during the month of Ramadan, especially (i) Boukhris et al. (2019a) who reported that N45 was the better nap opportunity duration (i.e., compared to 25 and 35 min) for improving performance and reducing RPE scores during the 5-m shuttle run test and (ii) Hsouna et al. (2019a) who reported that naps ≥ 35 min had better results for improving physical performance and attention. Hammouda et al. (2018) reported that the highest power, the lowest power, and the mean power during a running-based anaerobic sprint test was higher after a 90-min nap than after a 20-min nap. Also, Boukhris et al. (2020a) reported that a 90-min nap was better than a 40-min nap for enhancing physical performance and reducing fatigue perception. It has been suggested that a long afternoon nap may be comparable to a sleep night in terms of sleep quality (Jiang et al., 2018). Also, it has been suggested that the improvement in physical performance after longer naps of 40–90 min could be explained by the greater amount of time spent in slow wave sleep, which is important for recovery of daily metabolism (Mulrine et al., 2012). Therefore, in the present study, the greater increases in performance during the 5-m shuttle run test observed during N45 condition at ER might be due to the role of the slow wave sleep (Boukhris et al., 2019a). In addition to that, the RPE mean scores during the 5m shuttle run test were significantly lower after N45 in comparison to NN and N25 during all periods of the study which could explain the increases in performance during this short-term maximal exercise (Boukhris et al., 2019a). In support of this idea, FI recorded during the 5-m shuttle run test were significantly correlated with RPE values. More importantly, it has been reported that an enhancement of performance during the 5 m shuttle run tests could be explained by (i) a decrease of muscle damage and inflammation, and (ii) an improvement of perceived recovery, exertion, and muscle soreness during and/or following the 5-m shuttle run test (Boukhris et al., 2021).

Studies such as the present one are subject to some limitations, such as the lack of objective sleep measurement during the nap time (e.g., by the use of actimetry or polysomnography). However, actimetry is not effective to evaluate a short nap because it estimates sleep time by recording the movements of the body; in a short nap, it is possible that the participant does not move but without having slept. Although polysomnography may provide a truer picture of actual sleep architecture, the equipment may affect participants’ sleep for these short-period naps. To overcome this, previous studies have used a questionnaire to quantify participants’ sleepiness (Waterhouse et al., 2007; Boukhris et al., 2020b). Thus, the results of the present study must be confirmed by other studies while checking the states of sleepiness of the participants.

Another potential limitation is that the presentation of the testing periods cannot be randomized, and the order is BR, ER, AR for all participants. Possible effects of prior testing and the effects of external conditions for the different testing periods cannot be identified. Prior testing may generate positive learning effects, if the participants become more skilled at shuttle running, or negative effects, if the testing is no longer novel and exciting. In the present study, the ER testing occurred when external conditions (daily temperature and relative humidity) were more oppressive than in the BR testing. In addition, in 2016, when the data were collected, ER coincide with the summer solstice, meaning the day length was at its greatest (and the opportunity for sleep at its smallest) both in comparison to the BR and AR periods as well as in comparison to other years. The timing of Ramadan in 2016 perhaps provided the greatest opportunity to observe the negative effect of its observance on exercise performance.

## Conclusion

Results of the present study showed that napping has a beneficial effect on physical performance and perceived exertion before, during, and after Ramadan. During the testing periods before and after Ramadan, nap duration had little effect on the magnitude of the improvements elicited by the afternoon naps. However, during Ramadan (the ER testing period) 45 min was a more effective afternoon nap duration for improving performance and reducing RPE scores during the 5-m shuttle run test. Taken together, the present findings and results of previous studies may provide justifications for introducing nap periods into daily athletic training (Davies et al., 2010) and on the day of competition (O’Donnell et al., 2018). The longer nap is suggested for situations when the prior night’s sleep has been disrupted. The results definitely confirm the efficacy of napping as a tool to improve physical performance during Ramadan fasting. Future investigations may involve repeated naps with habitual/non-habitual nappers during a micro-cycle to explore the chronic effect of napping on physical and cognitive performance.

## Data Availability Statement

The raw data supporting the conclusions of this article will be made available by the authors, without undue reservation.

## Ethics Statement

The studies involving human participants were reviewed and approved by Local Institutional Research Ethics Committee (CPP: 0098/2018). The patients/participants provided their written informed consent to participate in this study.

## Author Contributions

OB, AA, KT, and HC contributed to conception and design of the study. OB wrote the first draft of the manuscript. All authors contributed to manuscript revision, read, and approved the submitted version.

## Conflict of Interest

The authors declare that the research was conducted in the absence of any commercial or financial relationships that could be construed as a potential conflict of interest. The reviewer OH declared a shared affiliation with one of the authors AA to the handling editor at the time of review.

## Publisher’s Note

All claims expressed in this article are solely those of the authors and do not necessarily represent those of their affiliated organizations, or those of the publisher, the editors and the reviewers. Any product that may be evaluated in this article, or claim that may be made by its manufacturer, is not guaranteed or endorsed by the publisher.

## References

[B1] Abdessalem R. Boukhris O. Hsouna H. Trabelsi K. Ammar A. Taheri M. (2019). Effect of napping opportunity at different times of day on vigilance and shuttle run performance. *Chronobiol. Int.* 36 1334–1342. 10.1080/07420528.2019.1642908 31368367

[B2] Abedelmalek S. Denguezli M. Chtourou H. Souissi N. Tabka Z. (2015). Does Ramadan fasting affect acylated ghrelin and growth hormone concentrations during short-term maximal exercise in the afternoon? *Biol. Rhythm Res.* 46 691–701.

[B3] Aloui A. Baklouti H. Souissi N. Chtourou H. (2019). Effects of Ramadan fasting on body composition in athletes: a systematic review effets du jeûne du Ramadan sur la composition corporelle des sportifs: revue systématique. *Tunis Med*. 97 1087–1094. 31691936

[B4] Aloui A. Chaouachi A. Chtourou H. Wong D. P. Haddad M. Chamari K. (2013). Effects of Ramadan on the diurnal variations of repeated-sprint performance. *Int. J. Sports Physiol. Perform*. 8 254–263. 10.1123/ijspp.8.4.350a 22952200

[B5] Aziz A. R. Che Muhamed A. M. Ooi C. H. Singh R. Chia M. Y. H. (2018). Effects of Ramadan fasting on the physical activity profile of trained Muslim soccer players during a 90-minute match. *Sci. Med. Footb*. 2 29–38.

[B6] Aziz A. R. Chia M. Y. H. Low C. Y. Slater G. J. Png W. Teh K. C. (2012). Conducting an acute intense interval exercise session during the Ramadan fasting month: what is the optimal time of the day? *Chronobiol. Int.* 29 1139–1150. 10.3109/07420528.2012.708375 22947072

[B7] Aziz A. R. Wahid M. F. Png W. Jesuvadian C. V. (2010). Effects of Ramadan fasting on 60 min of endurance running performance in moderately trained men. *Br. J. Sports Med.* 44 516–521. 10.1136/bjsm.2009.070425 20519256

[B8] BaHammam A. (2003). Sleep pattern, daytime sleepiness, and eating habits during the month of Ramadan. *Sleep Hypn*. 5 165–174.

[B9] Beck T. W. (2013). The importance of a priori sample size estimation in strength and conditioning research. *J. Strength Cond. Res*. 27 2323–2337. 10.1519/JSC.0b013e318278eea0 23880657

[B10] Blanchfield A. W. Lewis-Jones T. M. Wignall J. R. Roberts J. B. Oliver S. J. (2018). The influence of an afternoon nap on the endurance performance of trained runners. *Eur. J. Sport Sci*. 18 1177–1184. 10.1080/17461391.2018.1477180 29851569

[B11] Boddington M. K. Lambert M. I. Gibson A. S. C. Noakes T. D. (2001). Reliability of a 5-m multiple shuttle test. *J. Sports Sci.* 19 223–228. 10.1080/026404101750095394 11256826

[B12] Botonis P. G. Koutouvakis N. Toubekis A. G. (2021). The impact of daytime napping on athletic performance – A narrative review. *Scand. J. Med. Sci. Sports* 31, 2164–2177. 10.1111/sms.14060 34559915

[B13] Bougard C. Moussay S. Gauthier A. Espié S. Davenne D. (2009). Effects of waking time and breakfast intake prior to evaluation of psychomotor performance in the early morning. *Chronobiol. Int.* 26 324–336. 10.1080/07420520902774540 19212844

[B14] Boukhris O. Trabelsi K. Ammar A. Abdessalem R. Hsouna H. Glenn J. M. (2020a). A 90 min daytime nap opportunity is better than 40 min for cognitive and physical performance. *Int. J. Environ. Res. Public Health* 17:4650. 10.3390/ijerph17134650 32605240 PMC7369743

[B15] Boukhris O. Trabelsi K. Abdessalem R. Hsouna H. Ammar A. Glenn J. M. (2020b). Effects of the 5-m shuttle run test on markers of muscle damage, inflammation, and fatigue in healthy male athletes. *Int. J. Environ. Res. Public Health* 17:4375. 10.3390/ijerph17124375 32570815 PMC7344466

[B16] Boukhris O. Trabelsi K. Ammar A. Hsouna H. Abdessalem R. Altmann S. (2021). Performance, muscle damage, and inflammatory responses to repeated high-intensity exercise following a 40-min nap. *Res. Sports Med.* 1–18. Online ahead of print, 10.1080/15438627.2021.1988951 34665981

[B17] Boukhris O. Trabelsi K. Chtourou H. (2018). Evolution of dietary intake between before, during and after Ramadan observance in Tunisian physically active men: a systematic review. *Int. J. Sport Stud. Health* 1:e83782.

[B18] Boukhris O. Abdessalem R. Ammar A. Hsouna H. Trabelsi K. Engel F. A. (2019a). Nap opportunity during the daytime affects performance and perceived exertion in 5-m shuttle run test. *Front. Physiol.* 10:779. 10.3389/fphys.2019.00779 31281263 PMC6596336

[B19] Boukhris O. Trabelsi K. Shephard R. J. Hsouna H. Abdessalem R. Chtourou L. (2019b). Sleep patterns, alertness, dietary intake, muscle soreness, fatigue, and mental stress recorded before, during and after Ramadan observance. *Sports* 7:118. 10.3390/sports7050118 31109004 PMC6571812

[B20] Brisswalter J. Bouhlel E. Falola J. M. Abbiss C. R. Vallier J. M. Hauswirth C. (2011). Effects of Ramadan intermittent fasting on middle-distance running performance in well-trained runners. *Clin. J. Sport Med.* 21 422–427. 10.1097/JSM.0b013e3182293891 21857506

[B21] Brooks A. Lack L. (2006). A brief afternoon nap following nocturnal sleep restriction: which nap duration is most recuperative? *Sleep* 29 831–840. 10.1093/sleep/29.6.831 16796222

[B22] Buysse D. J. Reynolds C. F. III Monk T. H. Berman S. R. Kupfer D. J. (1989). The Pittsburgh sleep quality index: a new instrument for psychiatric practice and research. *Psychiatry Res*. 28 193–213. 10.1016/0165-1781(89)90047-4 2748771

[B23] Chamari K. Briki W. Farooq A. Patrick T. Belfekih T. Herrera C. P. (2016). Impact of Ramadan intermittent fasting on cognitive function in trained cyclists: a pilot study. *Biol. Sport* 33:49. 10.5604/20831862.1185888 26985134 PMC4786586

[B24] Chamari K. Roussi M. Bragazzi N. Chaouachi A. Abdul R. (2019). Optimizing training and competition during the month of Ramadan: recommendations for a holistic and personalized approach for the fasting athletes. *Tunis Med*. 97 1095–1103. 31691937

[B25] Chaouachi A. Chamari K. Roky R. Wong P. Mbazaa A. Bartagi Z. (2008). Lipid profiles of judo athletes during Ramadan. *Int. J. Sports Med.* 29 282–288. 10.1055/s-2007-965338 17879887

[B26] Chaouachi A. Coutts A. J. Chamari K. Wong D. P. Chaouachi M. Chtara M. (2009). Effect of Ramadan intermittent fasting on aerobic and anaerobic performance and perception of fatigue in male elite judo athletes. *J. Strength Cond. Res.* 23 2702–2709. 10.1519/JSC.0b013e3181bc17fc 19910805

[B27] Chtourou H. H’mida C. Boukhris O. Trabelsi K. Ammar A. Souissi N. (2019a). Nap opportunity as a strategy to improve short-term repetitive maximal performance during the 5-m shuttle run test: a brief review. *Int. J. Sport Stud. Health* 2:e97538.

[B28] Chtourou H. Trabelsi K. Boukhris O. Ammar A. Shephard R. J. Bragazzi N. L. (2019b). Effects of Ramadan fasting on physical performances in soccer players: a systematic review effets du jeûne de Ramadan sur les performances physiques des footballeurs: revue systématique. *Tunis Med*. 97 1114–1131. 31691939

[B29] Chtourou H. Hammouda O. Chaouachi A. Chamari K. Souissi N. (2012). The effect of time-of-day and Ramadan fasting on anaerobic performances. *Int. J. Sports Med.* 33 142–147. 10.1055/s-0031-1286251 22318530

[B30] Chtourou H. Hammouda O. Souissi H. Chamari K. Chaouachi A. Souissi N. (2011). The effect of Ramadan fasting on physical performances, mood state and perceived exertion in young footballers. *Asian J. Sports Med.* 2:177. 10.5812/asjsm.34757 22375237 PMC3289213

[B31] Daaloul H. Souissi N. Davenne D. (2019). Effects of napping on alertness, cognitive, and physical outcomes of karate athletes. *Med. Sci. Sports Exerc*. 51 338–345. 30239491 10.1249/MSS.0000000000001786

[B32] Davies D. J. Graham K. S. Chow C. M. (2010). The effect of prior endurance training on nap sleep patterns. *Int. J. Sports Physiol. Perform*. 5 87–97. 10.1123/ijspp.5.1.87 20308699

[B33] Faul F. Erdfelder E. Lang A. G. Buchner A. (2007). G* Power 3: a flexible statistical power analysis program for the social, behavioral, and biomedical sciences. *Behav. Res. Methods* 39 175–191. 10.3758/bf03193146 17695343

[B34] Field A. P. (2014). *Kendall’s Coefficient of Concordance, Wiley StatsRef: Statistics Reference Online.* London: Encyclopedia ofStatistics in Behavioral Science.

[B35] Fullagar H. H. Skorski S. Duffield R. Hammes D. Coutts A. J. Meyer T. (2015). Sleep and athletic performance: the effects of sleep loss on exercise performance, and physiological and cognitive responses to exercise. *Sports Med.* 45 161–186. 10.1007/s40279-014-0260-0 25315456

[B36] Girard O. Farooq A. (2012). Effects of Ramadan fasting on repeated sprint ability in young children. *Sci. Sports* 27 237–240.

[B37] Haddad M. Chaouachi A. Castagna C. Hue O. Wong D. P. Tabben M. (2013). Validity and psychometric evaluation of the French version of RPE scale in young fit males when monitoring training loads. *Sci. Sports* 28 e29–e35.

[B38] Hammouda O. Chtourou H. Aloui A. Chahed H. Kallel C. Miled A. (2013). Concomitant effects of Ramadan fasting and time-of-day on apolipoprotein AI, B, Lp-a and homocysteine responses during aerobic exercise in Tunisian soccer players. *PLoS One* 8:e79873. 10.1371/journal.pone.0079873 24244572 PMC3823586

[B39] Hammouda O. Chtourou H. Aloui A. Mejri M. A. Chahed H. Miled A. (2014). Does Ramadan fasting affect the diurnal variations in metabolic responses and total antioxidant capacity during exercise in young soccer players? *Sport Sci. Health* 10 97–104.

[B40] Hammouda O. Chtourou H. Farjallah M. A. Davenne D. Souissi N. (2012). The effect of Ramadan fasting on the diurnal variations in aerobic and anaerobic performances in Tunisian youth soccer players. *Biol. Rhythm Res.* 43 177–190.

[B41] Hammouda O. Romdhani M. Chaabouni Y. Mahdouani K. Driss T. Souissi N. (2018). Diurnal napping after partial sleep deprivation affected hematological and biochemical responses during repeated sprint. *Biol. Rhythm Res*. 49 927–939.

[B42] Hayashi M. Hori T. (1998). The effects of a 20-min nap before post-lunch dip. *Sport Sci. Health* 52 203–204. 10.1111/j.1440-1819.1998.tb01031.x 9628152

[B43] Hayashi M. Ito S. Hori T. (1999). The effects of a 20-min nap at noon on sleepiness, performance and EEG activity. *Int. J. Psychophysiol*. 32 173–180. 10.1016/s0167-8760(99)00009-4 10380949

[B44] Herrera C. P. (2012). Total sleep time in Muslim football players is reduced during Ramadan: a pilot study on the standardized assessment of subjective sleep–wake patterns in athletes. *J. Sports Sci*. 30 S85–S91. 10.1080/02640414.2012.676666 22489547

[B45] Horne J. Reyner L. (2001). Sleep-related vehicle accidents: some guides for road safety policies. *Transp. Res. F Traffic Psychol. Behav*. 4 63–74. 10.1016/s1369-8478(01)00014-6

[B46] Hsouna H. Boukhris O. Abdessalem R. Trabelsi K. Ammar A. Shephard R. J. (2019a). Effect of different nap opportunity durations on short-term maximal performance, attention, feelings, muscle soreness, fatigue, stress and sleep. *Physiol. Behav.* 211:112673. 10.1016/j.physbeh.2019.112673 31491444

[B47] Hsouna H. Abdessalem R. Boukhris O. Trabelsi K. Chtourou L. Tahri N. (2019b). Short-term maximal performance, alertness, dietary intake, sleep pattern and mood states of physically active young men before, during and after Ramadan observance. *PLoS One* 14:e0217851. 10.1371/journal.pone.0217851 31163075 PMC6548427

[B48] Hsouna H. Boukhris O. Trabelsi K. Abdessalem R. Ammar A. Irandoust K. (2020). Effects of 25-min nap opportunity during Ramadan observance on the 5-m shuttle run performance and the perception of fatigue in physically active men. *Int. J. Environ. Res. Public Health* 17:3135. 10.3390/ijerph17093135 32365914 PMC7246774

[B49] Jiang F. Kobayashi T. Ichihashi T. Nomura S. (2018). Effect of a relatively long afternoon nap on autonomous nervous activity, sleep architecture, and subjective sleep quality. *IEEJ Trans. Electr. Electron. Eng.* 13 1357–1361.

[B50] Karli U. Guvenc A. Aslan A. Hazir T. Acikada C. (2007). Influence of Ramadan fasting on anaerobic performance and recovery following short time high intensity exercise. *J. Sports Sci. Med.* 6:490. 24149483 PMC3794490

[B51] Keramidas M. E. Siebenmann C. Norrbrand L. Gadefors M. Eiken O. (2018). A brief pre-exercise nap may alleviate physical performance impairments induced by short-term sustained operations with partial sleep deprivation–A field-based study. *Chronobiol. Int.* 35 1464–1470. 10.1080/07420528.2018.1490316 29985669

[B52] Kölling S. Duffield R. Erlacher D. Venter R. Halson S. L. (2019). Sleep-related issues for recovery and performance in athletes. *Int. J. Sports Physiol. Perform.* 14 144–148. 10.1123/ijspp.2017-0746 29651858

[B53] Leiper J. B. Junge A. Maughan R. J. Zerguini Y. Dvorak J. (2008). Alteration of subjective feelings in football players undertaking their usual training and match schedule during the Ramadan fast. *J. Sports Sci.* 26 S55–S69. 10.1080/02640410802538176 19085453

[B54] Mah C. D. Mah K. E. Kezirian E. J. Dement W. C. (2011). The effects of sleep extension on the athletic performance of collegiate basketball players. *Sleep* 34 943–950. 10.5665/SLEEP.1132 21731144 PMC3119836

[B55] Milner C. E. Fogel S. M. Cote K. A. (2006). Habitual napping moderates motor performance improvements following a short daytime nap. *Biol. Psychol.* 73 141–156. 10.1016/j.biopsycho.2006.01.015 16540232

[B56] Mulrine H. M. Signal T. L. Berg M. J. V. D. Gander P. H. (2012). Post-sleep inertia performance benefits of longer naps in simulated nightwork and extended operations. *Chronobiol. Int.* 29 1249–1257. 10.3109/07420528.2012.719957 23002951

[B57] O’Connor R. M. Rogers N. L. Van Dongen H. Dinges D. F. (2004). Dose response effects of short duration naps during extended wakefulness. *Sleep* 27 155–156.

[B58] O’Donnell S. Beaven C. M. Driller M. (2018). The influence of Match-Day napping in elite female Netball athletes. *Int. J. Sports Physiol. Perform.* 13 1143–1148. 10.1123/ijspp.2017-0793 29543074

[B59] Petit E. Mougin F. Bourdin H. Tio G. Haffen E. (2014). A 20-min nap in athletes changes subsequent sleep architecture but does not alter physical performances after normal sleep or 5-h phase-advance conditions. *Eur. J. Appl. Physiol.* 114 305–315. 24276580 10.1007/s00421-013-2776-7

[B60] Qasrawi S. O. Pandi-Perumal S. R. BaHammam A. S. (2017). The effect of intermittent fasting during Ramadan on sleep, sleepiness, cognitive function, and circadian rhythm. *Sleep Breath.* 21 577–586. 10.1007/s11325-017-1473-x 28190167

[B61] Reilly T. Waterhouse J. (2007). Altered sleep–wake cycles and food intake: the Ramadan model. *Physiol. Behav.* 90 219–228. 10.1016/j.physbeh.2006.09.004 17081572

[B62] Roky R. Herrera C. P. Ahmed Q. (2012). Sleep in athletes and the effects of Ramadan. *J. Sports Sci.* 30 S75–S84. 10.1080/02640414.2012.693622 22694752

[B63] Roky R. Houti I. Moussamih S. Qotbi S. Aadil N. (2004). Physiological and chronobiological changes during Ramadan intermittent fasting. *Ann. Nutr. Metab.* 48 296–303. 10.1159/000081076 15452402

[B64] Romdhani M. Dergaa I. Moussa-Chamari I. Souissi N. Chaabouni Y. Mahdouani K. (2021). The effect of post-lunch napping on mood, reaction time, and antioxidant defense during repeated sprint exercice. *Biol. Sport*. 38 629–638. 10.5114/biolsport.2021.103569 34937973 PMC8670803

[B65] Romdhani M. Hammouda O. Chaabouni Y. Mahdouani K. Driss T. Chamari K. (2019). Sleep deprivation affects post-lunch dip performances, biomarkers of muscle damage and antioxidant status. *Biol. Sport* 36:55. 10.5114/biolsport.2018.78907 30899140 PMC6413570

[B66] Romdhani M. Souissi N. Chaabouni Y. Mahdouani K. Driss T. Chamari K. (2020). Improved physical performance and decreased muscular and oxidative damage with Postlunch napping after partial sleep deprivation in athletes. *Int. J. Sports Physiol. Perform.* 15 874–883. 10.1123/ijspp.2019-0308 32023544

[B67] Samuels C. (2008). Sleep, recovery, and performance: the new frontier in high-performance athletics. *Neurol. Clin.* 26 169–180. 18295089 10.1016/j.ncl.2007.11.012

[B68] Shephard R. J. (2012). The impact of Ramadan observance upon athletic performance. *Nutrients* 4 491–505. 10.3390/nu4060491 22822448 PMC3397348

[B69] Souabni M. Hammouda O. Romdhani M. Trabelsi K. Ammar A. Driss T. (2021). Benefits of daytime napping opportunity on physical and cognitive performances in physically active participants: a systematic review. *Sports Med.* 51 2115–2146. 10.1007/s40279-021-01482-1 34043185

[B70] Souissi N. Souissi H. Sahli S. Tabka Z. Dogui M. Ati J. (2007). Effect of Ramadan on the diurnal variation in short-term high power output. *Chronobiol. Int.* 24 991–1007. 10.1080/07420520701661914 17994351

[B71] Suleiman K. H. Yates B. C. Berger A. M. Pozehl B. And Meza J. (2010). Translating the Pittsburgh sleep quality index into Arabic. *West. J. Nurs. Res.* 32 250–268. 10.1177/0193945909348230 19915205

[B72] Suppiah H. T. Low C. Y. Choong G. Chia M. (2019). Effects of a short daytime nap on shooting and sprint performance in high-level adolescent athletes. *Int. J. Sports Physiol. Perform.* 14 76–82. 10.1123/ijspp.2018-0107 29893599

[B73] Thorpe R. Sunderland C. (2012). Muscle damage, endocrine, and immune marker response to a soccer match. *J. Strength Cond. Res.* 26 2783–2790. 10.1519/JSC.0b013e318241e174 22124357

[B74] Trabelsi K. Bragazzi N. Zlitni S. Khacharem A. Boukhris O. El-Abed K. (2020a). Observing Ramadan and sleep-wake patterns in athletes: a systematic review, meta-analysis and meta-regression. *Br. J. Sports Med.* 54 674–680. 10.1136/bjsports-2018-099898 31154342

[B75] Trabelsi K. Ammar A. Boukhris O. Glenn J. M. Bott N. Stannard S. R. (2020b). Effects of Ramadan observance on dietary intake and body composition of adolescent athletes: systematic review and meta-analysis. *Nutrients* 12:1574. 10.3390/nu12061574 32481549 PMC7353054

[B76] Trabelsi K. Ammar A. Boukhris O. Glenn J. M. Clark C. C. T. Stannard S. R. (2022). Dietary intake and body composition during Ramadan in athletes: a systematic review and meta-analysis with meta-regression. *J. Am. Coll. Nutr.* 10.1080/07315724.2021.2000902 35512756

[B77] Trabelsi K. Ammar A. Glenn J. M. Boukhris O. Khacharem A. Bouaziz B. (2021). Does observance of Ramadan affect sleep in athletes and physically active individuals? A systematic review and meta-analysis. *J. Sleep Res.* Online ahead of print, 10.1111/jsr.13503 34693577

[B78] Trabelsi K. El Abed K. Stannard S. R. Jammoussi K. Zeghal K. M. Hakim A. (2012a). Effects of fed-versus fasted-state aerobic training during Ramadan on body composition and some metabolic parameters in physically active men. *Int. J. Sport Nutr. Exerc. Metab.* 22 11–18. 10.1123/ijsnem.22.1.11 22248495

[B79] Trabelsi K. Stannard S. R. Maughan R. J. Jammoussi K. Zeghal K. Hakim A. (2012b). Effect of resistance training during Ramadan on body composition and markers of renal function, metabolism, inflammation, and immunity in recreational bodybuilders. *Int. J. Sport Nutr. Exerc. Metab.* 22 267–275. 10.1123/ijsnem.22.4.267 22855788

[B80] Verweij I. M. Onuki Y. Van Someren E. J. Van der Werf Y. D. (2016). Sleep to the beat: a nap favours consolidation of timing. *Behav. Neurosci.* 130:298. 10.1037/bne0000146 27214501

[B81] Waterhouse J. Alkib L. Reilly T. (2008). Effects of Ramadan upon fluid and food intake, fatigue, and physical, mental, and social activities: a comparison between the UK and Libya. *Chronobiol. Int.* 25 697–724. 10.1080/07420520802397301 18780199

[B82] Waterhouse J. Atkinson G. Edwards B. Reilly T. (2007). The role of a short post-lunch nap in improving cognitive, motor, and sprint performance in participants with partial sleep deprivation. *J. Sports Sci.* 25 1557–1566. 10.1080/02640410701244983 17852691

[B83] Zerguini Y. Kirkendall D. Junge A. Dvorak J. (2007). Impact of Ramadan on physical performance in professional soccer players. *Br. J. Sports Med.* 41 398–400. 10.1136/bjsm.2006.032037 17224435 PMC2465333

